# The Value of Cognitive and Physical Function Tests in Predicting Falls in Older Adults: A Prospective Study

**DOI:** 10.3389/fmed.2022.900488

**Published:** 2022-07-05

**Authors:** Rong Zhou, Jiayu Li, Meiling Chen

**Affiliations:** ^1^The Second School of Clinical Medicine, Zhejiang Chinese Medical University, Hangzhou, China; ^2^School of Humanities and Management, Zhejiang Chinese Medical University, Hangzhou, China

**Keywords:** balance, cognition, fall risk assessment, grip strength, older adults, walking speed, the five times sit-to-stand test

## Abstract

**Introduction:**

Previous studies suggested that physical and cognitive function can be indicators to assess the risk of falls in the elderly. Various tests are widely used in geriatric clinical studies as assessment tools of physical and cognitive function. However, large sample studies comparing the fall predictive value of these tests are still sparse. This study was conducted to investigate the value of cognitive and physical function tests in predicting the risk of subsequent falls in the elderly, with the overarching goal of providing more evidence on fall-risk assessment.

**Methods:**

The current study was based on the data of respondents aged 60 and above from the China Health and Retirement Longitudinal Study (CHARLS). Data from the 2015 CHARLS national survey were used as the baseline data, and the fall data in 2018 were used as the follow-up data. Physical function tests included balance, walking speed, the five times sit-to-stand test (FTSST), and grip strength. The value of cognitive and physical function tests in predicting falls was evaluated by logistic regression analysis and receiver operating characteristic (ROC) curves.

**Results:**

The incidence of falls among the 4,857 subjects included in this study was 20.86%. Results showed that cognition (OR = 0.83, 95% CI: 0.70–0.98), the FTSST (OR = 3.51, 95% CI: 1.66–7.46), and grip strength (OR = 1.02, 95% CI: 1.01–1.03) were independent predictors of falls in the full sample after adjusting for various confounders. Notably, the above tests showed better predictive value for falls for the oldest-old (≥ 80 years) subjects.

**Conclusion:**

Overall, results showed that grip strength, the FTSST, and cognition tests are simple and practicable tools for identifying individuals at higher risk of falls in the community. Moreover, the fall predictive performance of physical and cognitive function tests was age-dependent, with a higher predictive value in older adults aged 80 and above.

## Introduction

Falls are common among the elderly. It is reported that the annual incidence of accidental falls among older individuals is 28–35%, and the incidence of falls increases with age (1). Falls can lead to pain, fracture, disability, decreased ability to perform activities of daily living, and even death (2–5). With the irreversible trend of global population aging, the prevalence of falls and fall-related costs of older adults are increasing annually (6). Therefore, it is of great significance to carry out a fall risk assessment for the elderly, with the ultimate goal of preventing the occurrence of fall events.

Declines in physical and cognitive function in older adults tend to be associated with an increased likelihood of adverse health events (7). Many studies have revealed that impairments in domains of physical and cognitive function are strong risk factors for falls among the elderly (8–11). In addition, recent studies have demonstrated a strong correlation between physical and cognitive function in aging (12), with a combination of physical and cognitive function deficits being more likely to result in adverse outcomes, including falls, than physical or cognitive deficits alone (13).

A previous study proposed that performance-based measures may be more sensitive than self-reported measures in assessing physical and cognitive function (14). Given that balance, walking speed, and muscle strength are important indicators that reflect physical function (8), they have been commonly used in geriatric clinical studies. The five times sit-to-stand test (FTSST) and grip strength are two simple tools used to rapidly assess muscle strength. In addition, there is a wide range of cognitive screening tools in the clinic, among which the Mini-Mental State Examination (MMSE) is the most widely used (15). Notably, the above tests can be easily implemented and are suitable for use among the elderly in the community.

Numerous studies have shown that objective evaluation of physical and cognitive function is of great significance in predicting falls (16, 17). For instance, grip strength was proved to be the most significantly independent risk factor of falls in a recent study (18). The FTSST was also significantly associated with falls (19), and another study demonstrated that lower limb power assessed based on the FTSST is more predictive of falls than strength in older adults (20). Additionally, timed up and go test (TUGT) has been shown to have a higher predictive value of both falls and repeated falls compared with walking speed and grip strength (21). However, there are still few studies that have compared the value of both physical and cognitive function tests in predicting falls based on the same large sample and stable research methods (22). Moreover, the results on the fall predictive value of multiple function tests in previous studies have been quite inconsistent. Therefore, further research is still needed to better evaluate the fall predictive value of common tests in different aging stages.

In older adults aged 80 and above, the annual incidence of falls has increased to about 50% with increased difficulty in completing all the tests (23). Notably, physical performance is considered a strong predictor of mortality and disability in this age cohort (24). Based on this, subjects included were divided into two subgroups according to age: the young elderly (aged 60–79 years) and the oldest-old (aged ≥ 80 years). The value of the five tests in predicting falls was, respectively, analyzed in the two groups. This study aimed at investigating the fall prediction value of physical and cognitive function tests that are commonly used in geriatric clinical studies based on representative data from a nationwide study, with the overall goal of providing more insights into establishing a more accurate predictor of falls for this particular population.

## Materials and Methods

### Study Design and Participants

The data used in the current study was acquired from the Chinese Health and Retirement Longitudinal Survey (CHARLS). CHARLS is a nationally representative longitudinal survey conducted among middle-aged and older adults in China by Peking University. The survey includes assessments of basic demographics, health status, physical measurement, utilization of medical services, and socioeconomic status through one-on-one interviews based on a structured questionnaire. The clinical assessment of physical and cognitive function was carried out in the participants’ homes by interviewers recruited in advance. Centralized training and regular assessment were conducted for interviewers to ensure the authenticity and effectiveness of the collected data. By 2018, the survey had collected data from a total of 19,000 respondents in 12,400 households (25).

In the current study, the demographic data, lifestyle and health status data, grip strength, balance, walking speed, the FTSST, and cognition data were taken from the 2015 data. The subsequent fall data were obtained from the 2018 data as the outcome variable.

**FIGURE 1:**
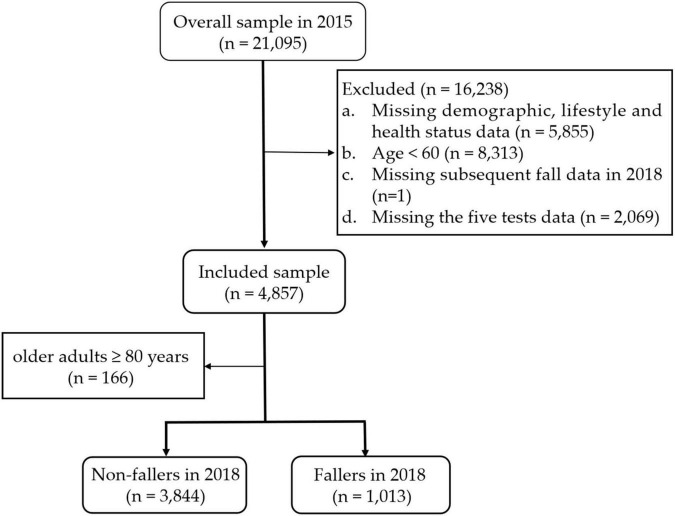
Flow-chart showing the process of enrolling participants in the analysis.

Participants aged 60 and older were selected for inclusion in this study. The exclusion criteria were as follows: (1) missing demographic, lifestyle behaviors and health status data; (2) aged < 60; (3) missing subsequent fall data in 2018; and (4) missing data for the five tests. Finally, a total of 4,857 individuals were included. Figure 1 shows the detailed exclusion process.

### Study Variables

#### Fall

In the CHARLS survey, participants were asked to answer “yes” or “no” to the question “Have you fallen in the last 2 years?” The participants included in this study were divided into the falls group and the no falls group according to their answers.

#### Cognition

Cognitive function was quantified in the CHARLS questionnaire with a full score of 21, including telephone interviews for cognitive status-10 (TICS-10), visual-spatial ability test, and episodic memory. TICS-10 is a reliable and valid method for MMSE, with a total score of 10 points. Participants were asked to answer the year, season, month, date, and the day of the week at that time, one point would be given for each correct answer. Then, participants needed to calculate 100 minus 7 for five consecutive times, with one point for each correct calculation. With regard to the visual-spatial ability, participants were asked to draw the picture provided, with one point being given for successfully drawing. Episodic memory was assessed through immediate and delayed words recall with a total score of 10. A list consisting of 10 words was provided by the interviewer, and the participants would be given one point for each correct word recall. The scores of the above tests were added to the total cognition score (26).

#### Balance

The balance test required participants to stand with the heel of one foot in front of the other, touching the toes of the other foot for 30/60 s (30 s for 70 years old or above and 60 s for less than 70 years old) without moving their feet. The result is either recorded as “pass” or “fail.”

#### Walking Speed

In the walking speed test, participants were asked to walk along a 2.5-m long line twice, and the average time of the two times was recorded.

#### Grip Strength

Grip strength was measured by a Yuejian™ WL-1000 dynamometer (Nantong Yuejian Physical Measurement Instrument Co., Ltd., Nantong, China). After the demonstration, the participants clenched the dynamometer as hard as they could, held it for a few seconds, and then released it. Each hand was tested twice, and the mean value of grip strengths (in kilograms) was recorded.

#### Five Times Sit-to-Stand Test

In the FTSST, participants were asked to stand up straight and then sit as fast as possible on a standard-height (43 cm) chair without armrests, five times without stopping or pushing with their arms. The result was recorded as either “pass” or “fail.”

### Controlled Variables

We selected the following covariates as potential risk factors for falls based on the relevant literature: (1) Demographics: including age, gender, marital status, and education level. Marital status was divided into two groups: “married with spouse present” group and “without a spouse present” group (including participants who were separated/divorced/widowed/never married/cohabitated/married but not living with spouse temporarily); (2) Behavior and lifestyle: smoking status was classified into “yes” or “no” according to the answer; sleep status included the night sleep time and whether a nap was usually taken (27); (3) Health status: CHARLS questionnaire investigated 14 chronic diseases. According to the answers to “Have you been diagnosed with [the chronic disease] by a doctor?,” participants were divided into two groups, one with chronic diseases (one chronic disease or more), and the other without. Disability variables included physical disabilities, intellectual disability, vision problems, hearing problems, and a speech impediment. In addition, the CES-D scale was applied to evaluate depression with a total score of 30. Scores ≥ 10 indicated the presence of obvious symptoms of depression (28, 29); (4) Fall history: participants were asked to answer “yes” or “no” to the question “Have you fallen since your last visit?”

### Statistical Analyses

The data in DTA format was obtained from the CHARLS database. Statistical analyses were performed using STATA MP 17.0 and SPSS 26.0. The above risk factors were included as covariates during the univariate analysis of falls, and the categorical variables were analyzed through chi-squared test. If the continuous variables conformed to a normal distribution or approximate normal distribution, the independent samples *t*-test was used; otherwise, a non-parametric rank-sum test of two independent samples was used. The associations among cognition, balance, walking speed, grip strength, FTSST, and falls were evaluated by stepwise forward logistic regression models, controlling for confounding factors. The receiver operating characteristic (ROC) curve were generated to determine the predictive value of the five tests. To explore the predictive value in different age cohorts, the above analysis was first conducted in the full sample and then in older adults aged ≥ 80 years. A two-tailed *p*-value < 0.05 was considered statistically significant.

## Results

Among the 4,857 subjects enrolled in this study, 2,667 (54.91%) were men and 2,190 (45.09%) were women. The age of the subjects ranged from 60 to 102 years, with a mean age of 67.16 ± 5.78. Results showed that 1,013 (20.86%) people had fallen in the previous 2 years.

According to the follow-up time, respectively published in both data sets, the mean duration between the two assessment points for participants included in this study was 35.98 ± 0.74 months, with the shortest of 31 months, and the longest of 38 months.

### General Characteristics of the Subjects

The baseline characteristics of the falls group and the no falls group were shown in Table 1. Results showed that gender was significantly associated with falls (χ^2^ = 51.64, *p* < 0.001) with a higher risk of falls in females than in males. Increased age was significantly associated with a higher possibility of falls (χ^2^ = 4.55, *p* < 0.001). Older adults with lower educational level were more likely to suffer from falls (χ^2^ = 23.25, *p* < 0.001). And older adults who did not have a spouse present had a higher risk of falls (χ^2^ = 13.80, *p* < 0.001).

**TABLE 1:** Comparison of general characteristics between the falls and no falls group (*n* = 4,857).

Variables	Total	Fall		χ^2^/t	*P*-value
		Yes	No		
Gender, *n* (%)				51.64	<0.001***
Male	2,667 (54.91)	455 (17.06)	2,212 (82.94)		
Female	2,190 (45.09)	558 (25.48)	1,632 (74.52)		
Age		67.90 ± 6.06	66.97 ± 5.69	4.55	<0.001***
Marital status, *n* (%)				13.80	<0.001***
Married with spouse present	3,933 (80.98)	779 (19.81)	3,154 (80.19)		
Without a spouse present	924 (19.02)	234 (25.32)	690 (74.68)		
Education, *n* (%)				23.25	<0.001***
Illiteracy	1,102 (22.69)	285 (25.86)	817 (74.14)		
Primary school or below	2,573 (52.98)	512 (19.90)	2,061 (80.10)		
Middle school	784 (16.14)	147 (18.75)	637 (81.25)		
High school or above	398 (8.19)	69 (17.34)	329 (82.66)		
Smoking, *n* (%)				10.68	0.001**
Yes	1,762 (36.28)	323 (18.33)	1,439 (81.67)		
No	3,095 (63.72)	690 (22.29)	2,405 (77.71)		
Physical disabilities, *n* (%)				28.38	<0.001***
Yes	350 (7.21)	112 (32.00)	238 (68.00)		
No	4,507 (92.79)	901 (19.99)	3,606 (80.01)		
Intellectual disability, *n* (%)				14.28	<0.001***
Yes	331 (6.81)	96 (29.00)	235 (71.00)		
No	4,526 (93.19)	917 (20.26)	3,609 (79.74)		
Vision problems, *n* (%)				6.65	0.01*
Yes	724 (14.91)	177 (24.45)	547 (75.55)		
No	4,133 (85.09)	836 (20.23)	3,297 (79.77)		
Hearing problems, *n* (%)				8.97	0.003**
Yes	1,055 (21.72)	255 (24.17)	800 (75.83)		
No	3,802 (78.28)	758 (19.94)	3,044 (80.06)		
Speech impediment, *n* (%)				1.27	0.26
Yes	35 (0.72)	10 (28.57)	25 (71.43)		
No	4,822 (99.28)	1,003 (20.80)	3,819 (79.20)		
Night sleep duration (in hours), *n* (%)				45.28	<0.001***
<6	1,519 (31.27)	404 (26.60)	1,115 (73.40)		
6∼8	2,892 (59.54)	519 (17.95)	2,373 (82.05)		
> 8	446 (9.18)	90 (20.18)	356 (79.82)		
Napduration (in minutes), *n* (%)				10.88	0.012*
0	1,879 (38.69)	427 (22.72)	1,452 (77.28)		
<30	337 (6.94)	77 (22.85)	260 (77.15)		
30∼60	1,631 (33.58)	329 (20.17)	1,302 (79.83)		
> 60	1,010 (20.79)	180 (17.82)	830 (82.18)		
Chronic diseases, *n* (%)				10.40	<0.001***
No	1,099 (22.63)	191 (17.38)	908 (82.62)		
Yes	3,758 (77.37)	822 (21.87)	2,936 (78.13)		
CES-D score, *n* (%)				15.25	<0.001***
0–9	959 (19.74)	156 (16.27)	803 (83.73)		
10–30	3,898 (80.26)	857 (21.99)	3,041 (78.01)		
History of falls, *n* (%)				244.35	<0.001***
Yes	887 (18.26)	356 (40.14)	531 (59.86)		
No	3,970 (81.74)	657 (16.55)	3,313 (83.45)		

*p < 0.05, **p < 0.01, ***p < 0.001.

In addition, there were statistically significant differences in smoking status (χ^2^ = 10.68, *p* = 0.001), physical disabilities (χ^2^ = 28.38, *p* < 0.001), intellectual disability (χ^2^ = 14.28, *p* < 0.001), vision problems (χ^2^ = 6.65, *p* = 0.01), hearing problems (χ^2^ = 8.97, *p* = 0.003), night sleep duration (χ^2^ = 45.28, *p* < 0.001), nap duration (χ^2^ = 10.88, *p* = 0.012), chronic diseases (χ^2^ = 10.40, *p* < 0.001), depression (χ^2^ = 15.25, *p* < 0.001), and fall history (χ^2^ = 244.35, *p* < 0.001) between the falls group and the no falls group. However, there was no significant difference with regard to speech impediment.

### Characteristics of the Five Tests

The results of the five function tests were compared between the two groups of older adults. Table 2 shows that there were significant differences in balance (χ^2^ = 22.90, *p* < 0.001), grip strength (*t* = –4.40, *p* < 0.001), and cognition score (t = –6.19, *p* < 0.001) between the falls group and the no falls group. In addition, the difference in the FTSST was statistically significant (χ^2^ = 4.04, *p* = 0.044).

**TABLE 2:** Comparison of the five function tests between the falls and no falls group in the full sample (*n* = 4,857).

Variables	Total	Fall		χ^2^/t	*P*-value
		Yes	No		
Balance, *n* (%)				22.90	<0.001*
Pass	3,876 (79.80)	754 (19.45)	3,122 (80.55)		
Fail	981 (20.20)	259 (26.40)	722 (73.60)		
Walking speed (in seconds)		3.96 ± 15.69	3.41 ± 5.32	1.83	0.067
FTSST, *n* (%)				4.04	0.044*
Pass	4,826 (99.36)	1,002 (20.76)	3,824 (79.24)		
Fail	31 (0.64)	11 (35.48)	20 (64.52)		
Grip strength (kg)	27.85 ± 9.69	25.63 ± 9.33	29.20 ± 25.37	–4.40	<0.001***
Cognition		11.01 ± 3.94	11.82 ± 3.66	–6.19	<0.001***

*p < 0.05, **p < 0.01, ***p < 0.001.

### Correlations Between the Variables

Before binary logistic regression, the correlations between the variables were tested. According to the Pearson’s correlation test, the left-hand grip strength was significantly associated with the right hand, and thus we used the right-hand grip strength to represent the individual grip strength.

### Associations Between the Five Tests and Falls in the Full Sample

To further evaluate the associations between the above tests and falls, cognition, balance, walking speed, FTSST, and grip strength were added to five independent binary logistics models. Table 3 shows that cognition, grip strength, and FTSST were independent risk factors of falls after adjusting for confounding factors (including gender, age, marital status, education level, physical disabilities, intellectual disability, night sleep duration, depression, chronic disease, and history of falls). Participants with higher cognitive scores had a lower probability of falls compared to participants with lower cognitive scores (OR = 0.83, 95% CI: 0.70–0.98, *p* = 0.02). Moreover, older adults with lower grip strength were more likely to fall (OR = 1.02, 95% CI: 1.01–1.03, *p* < 0.001), and failing in FTSST was associated with a higher possibility of falls (OR = 3.51, 95% CI: 1.66–7.46, *p* = 0.001).

**TABLE 3:** Binary logistic regression models for falls in the full sample (*n* = 4,857).

	Model 1 (cognition)	*P*	Model 2 (balance)	*P*	Model 3 (walking speed)	*P*	Model 4 (FTSST)	*P*	Model 5 (grip strength)	*P*
	OR (95%CI)		OR (95%CI)		OR (95%CI)		OR (95%CI)		OR (95%CI)	
Gender	1.46 (1.25–1.71)	<0.001	1.45 (1.24–1.69)	<0.001	1.47 (1.26–1.71)	<0.001	1.48 (1.27–1.73)	<0.001	1.19 (1.00–1.42)	0.05
Age	1.01 (1.01–1.02)	<0.001	1.01 (1.01–1.02)	<0.001	1.01 (1.01–1.02)	<0.001	1.00 (0.99–1.01)	0.456	1.01 (1.00–1.01)	0.018
Marital status	1.35 (1.14–1.59)	<0.001	1.37 (1.15–1.62)	<0.001	1.36 (1.15–1.61)	<0.001	1.26 (1.05–1.50)	0.011	1.27 (1.07–1.51)	0.006
Education		0.191		0.227		0.222		0.383		0.32
Illiteracy (ref)										
Primary school or below	1.23 (0.90–1.67)	0.20	1.09 (0.82–1.46)	0.546	1.08 (0.81–1.45)	0.599	0.99 (0.73–1.33)	0.924	1.10 (0.82–1.50)	0.542
Middle School	1.33 (1.01–1.74)	0.04	1.26 (0.96–1.63)	0.092	1.25 (0.96–1.62)	0.1	1.14 (0.87–1.50)	0.344	1.23 (0.94–1.61)	0.128
High school or above	1.21 (0.89–1.64)	0.23	1.19 (0.88–1.61)	0.272	1.18 (0.87–1.60)	0.282	1.07 (0.78–1.46)	0.675	1.12 (0.82–1.52)	0.476
Physical disabilities	0.62 (0.48–0.80)	<0.001	0.62 (0.49–0.80)	<0.001	0.62 (0.48–0.80)	<0.001	0.61 (0.47–0.78)	<0.001	0.65 (0.51–0.84)	0.001
Intellectual disability	0.78 (0.60–1.01)	0.06	0.77 (0.59–1.01)	0.056	0.77 (0.59–1.00)	0.046	0.76 (0.58–0.99)	0.038	0.78 (0.60–1.02)	0.071
Night sleep duration (in hours)		0.011		0.008		0.009		0.008		0.011
<6 (ref)										
6~8	1.02 (0.79–1.32)	0.87	1.04 (0.81–1.33)	0.773	1.04 (0.81–1.33)	0.777	0.95 (0.74–1.24)	0.72	0.97 (0.75–1.25)	0.799
>8	1.36 (1.07–1.72)	0.01	1.39 (1.09–1.77)	0.007	1.39 (1.09–1.76)	0.008	1.27 (0.99–1.62)	0.062	1.27 (0.99–1.62)	0.057
CES–D score	1.27 (1.04–1.54)	0.02	1.27 (1.04–1.54)	0.018	1.26 (1.0–1.54)	0.019	1.26 (1.04–1.53)	0.021	1.24 (1.02–1.51)	0.03
Chronic diseases	1.15 (0.96–1.38)	0.13	1.15 (0.96–1.38)	0.124	1.15 (0.96–1.38)	0.126	1.14 (0.95–1.37)	0.16	1.13 (0.94–1.35)	0.204
History of falls	0.34 (0.29–0.40)	<0.001	0.34 (0.29–0.40)	<0.001	0.34 (0.29–0.40)	<0.001	0.34 (0.29–0.40)	<0.001	0.34 (0.29–0.40)	<0.001
Tests	0.83 (0.70–0.98)	0.02*	0.88 (0.74–1.05)	0.152	1.00 (0.99–1.00)	0.2	3.51 (1.66–7.46)	0.001**	1.02 (1.01–1.03)	<0.001***

*p < 0.05, **p < 0.01, ***p < 0.001. OR, odds ratio; 95% CI, 95% confidence interval. Adjusted covariates: gender, age, marital status, education, physical disabilities, intellectual disability, night sleep duration, depression, chronic disease, and history of falls.

**FIGURE 2:**
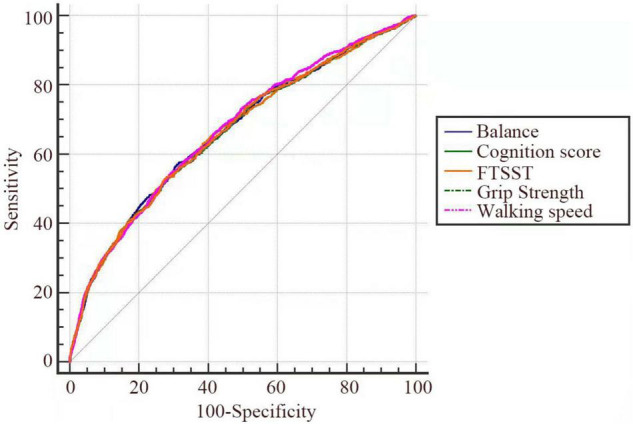
ROC curves of the five tests in the full sample (*n* = 4,857).

ROC curves were plotted to analyze the predictive value of the five tests for falls in the full sample (Figure 2). Table 4 displays the AUC of the five tests in the full sample.

**TABLE 4:** The AUC of the five tests in the full sample (*n* = 4,857).

Tests	AUC	95% CI	Sensitivity (%)	Specificity (%)	Youden index (%)	*p*
Grip strength	0.67	0.66–0.68	54.39	70.94	25.33	<0.001***
FTSST	0.66	0.65–0.68	52.42	72.79	25.21	<0.001***
Cognition	0.66	0.65–0.68	49.75	75.03	24.78	<0.001***
Balance	0.67	0.65–0.68	56.96	69.09	26.05	<0.001***
Walking speed	0.66	0.65–0.68	53.31	72.03	25.34	<0.001***

***p < 0.001. AUC, Area Under the Curve.

### The Predictive Value of the Five Tests in Older Adults Aged ≥ 80 Years

**FIGURE 3:**
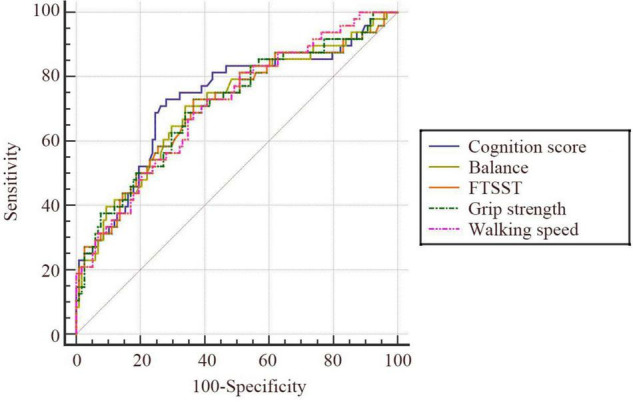
ROC curves of the five tests in older adults ≥ 80 years (*n* = 166).

Older adults aged 80 and above showed a higher incidence of falls and increased difficulty in completing all the tests. Based on this, the value of the five tests in predicting falls was, respectively analyzed in older adults aged ≥ 80 years. According to the ROC curve, the five tests showed better predictive value for falls in older adults aged 80 and above (Figure 3 and Table 5). Among these tests, cognition had the highest predictive value (AUC = 0.73, 95% CI: 0.65–0.79). The corresponding sensitivity and specificity values for cognition were 72.92 and 72.03%, respectively.

**TABLE 5:** The AUC of the five tests in older adults ≥ 80 years (*n* = 166).

Tests	AUC	95% CI	Sensitivity (%)	Specificity (%)	Youden index (%)	*p*
Grip strength	0.71	0.63–0.78	68.75	66.10	34.85	<0.001***
Cognition	0.73	0.65–0.79	72.92	72.03	44.95	<0.001***
Balance	0.71	0.64–0.78	70.83	66.10	36.94	<0.001***
Walking speed	0.71	0.63–0.78	68.75	63.56	32.31	<0.001***
FTSST	0.71	0.63–0.77	72.92	63.56	36.48	<0.001***

***p < 0.001. AUC, Area Under the Curve.

## Discussion

Population aging has led to the advent of a tremendous challenge: falls are not only the leading cause of injury and death among the elderly, but also pose a serious global health issue. Many previous studies have analyzed the predictive value of various function tests in falls (13, 17, 18, 21), but the results obtained in different studies were not consistent. The current study was conducted to compare the value of five physical and cognitive function tests in predicting falls based on a nationally representative survey. In this population-based study involving older adults, we evaluated the value of five clinically important function tests in predicting falls in different age cohorts.

China is facing the severe challenge of aging, and the high incidence of falls among older adults cannot be ignored. A recent study has found that the median annual incidence of falls among the elderly in China was 18% (30). Chu et al. demonstrated in their study that the prevalence of falls of the Chinese elderly were 19.3% (31). Consistently, the incidence of falls found in this study was 20.86%. In addition, previous studies have reported many risk factors for falls, including age, gender, history of falls, cognitive impairment, balance, chronic disease, and other factors (32–37). Results obtained in this study indicated that gender, age, marital status, education level, smoking, history of falls, disabilities, sleep duration, depression, and chronic diseases were significantly associated with falls, and balance instability, muscle weakness, as well as cognitive impairment in the five tests were independent risk factors of falls.

In the full sample analysis, poorer performance in the FTSST, cognition, and grip strength were significantly associated with an increased risk of falls after adjusting for various confounding factors, which was consistent with findings reported in previous studies (38–42). It is widely believed that a decline in cognitive function leads to various physiological and functional impairments involving gait (43, 44), balance (45), reaction time, and muscle strength (46), which increases the risk of falls. However, compared to physical function, age-related declines in cognitive function are generally not detected until much later (47), especially in the pre-dementia stage including mild cognitive impairment (MCI). Our results showed that cognition was a sensitive and specific index in predicting falls in the elderly. Collectively, our findings suggested that early identification of older adults with cognitive impairment may be an important strategy for reducing the risk of falls.

Given that the included subjects had a wide age distribution, we further evaluated the predictive value of the five tests in different age cohorts. The oldest-old (≥ 80 years) cohort is the most rapidly growing age group globally (48), and physical performance is considered as a strong predictor of mortality and disability in this group (24). Therefore, considering the further decline in physical function, fall prediction tools with higher accuracy are urgently needed for this cohort. Previous studies have evaluated the fall prediction value of different physical function tests, such as the Berg balance scale, Timed Up and Go test, and Tinetti balance scale (49, 50). However, for people in their 80s, especially when applied to large-scale fall risk screening in the community, the best tool should be simple and time-saving, taking into account its sensitivity and specificity. In the current study, the five tests showed better predictive value for falls in the oldest-old cohort. These tests require little space and do not need special equipment. Therefore, clinicians can easily use them to identify individuals at high risk of falls as they can easily be understood and executed by the elderly.

There is a strong association between physical and cognitive impairment, which was described as the “common cause” theory of aging in previous studies (47). Taylor et al. (46) proposed that the extent of cognitive impairment in the elderly may be quantified by monitoring their physical condition. Martin et al. (13) pointed out that the impact of physical deficits on falls can be amplified by cognitive impairment. Alcazar et al. (51) demonstrated a greater correlation between the sit-to-stand muscle strength tests and other risk factors (including physical and cognitive function) than the traditional FTSST in assessing muscle strength in the elderly, which may contribute to a more ideal tool for identifying the risk of falls in the elderly. Furthermore, Phirom et al. (52) proposed that simultaneous physical and cognitive training contributed to reducing the incidence of falls in seniors effectively. Thus, combining physical and cognitive impairments as a predictor may be helpful in fall-risk assessment, which is worth investigating in future studies.

There are several strengths of this study. First, we enrolled a large number of subjects (*n* = 4,857). The large sample size and the high quality of samples account for the representativeness of the study. Second, on the basis of evaluating the fall prediction values of different physical function tests, we also pay special attention to the impact of environmental factors and cognitive function on falls. The value of both physical and cognitive function tests in predicting falls were evaluated in this study based on the same large sample and stable research methods. It should be noted that the occurrence of falls results from the complex interaction between internal and external factors. It is expected that our findings will supplement the available evidence on the fall-risk assessment by providing data on appropriately large numbers of subjects of different age groups after adjusting for various potential confounding variables.

However, this study also had some limitations. First, we explored limited tests due to the shortage of data. Balance, walking speed, FTSST, and grip strength are only four among many physical function tests associated with falls. In addition, the evaluation of cognitive impairment in this study was based on questionnaire indicators in CHARLS. Since no other cognitive function evaluation scale was adopted, it may lead to evaluation bias in cognitive impairment and further affect the sensitivity and specificity of our results. Moreover, the study was conducted based on CHARLS data, thus, interpretation and promotion of our results should also cautiously consider the homogeneous ethnic background of the study population. Also, respondents in the CHARLS were expected to be able to answer the questions of the interviewers in line with the actual situation, which may lead to participant selection bias. As the data were mainly based on the respondents’ self-reports, the possibility of recall bias and self-selection bias cannot be completely excluded in this study. Finally, although we confirmed the predictive value of physical and cognitive function tests as independent predictors of falls, they showed a relatively low diagnostic significance (AUC < 0.7) in the full sample. Therefore, more efforts should be made to further explore how to improve the accuracy by combined prediction.

## Conclusion

In this study, nationally representative data were used to evaluate the value of five common physical and cognitive function tests in predicting the risk of subsequent falls in older adults, adding to evidence of the association of physical and cognitive deficits with falls. Results showed that grip strength, FTSST, and cognition tests may be simple and practicable tools for fall risk assessment in the community. Moreover, the five tests had better predictive value for falls in people aged 80 and older. Correspondingly, relevant strategies and measures should be put forward to identify individuals, especially the oldest-old, at high risk of falls in large-scale community screening.

## Data Availability Statement

The datasets for this study can be found in online repositories. The name of the repository and accession number can be found at: The China Health and Retirement Longitudinal Study (CHARLS), accessible on http://charls.pku.edu.cn/en. The current study is a secondary analysis of public data of CHARLS.

## Ethics Statement

The studies involving human participants were reviewed and approved by the Biomedical Ethics Review Committee of Peking University. The patients/participants provided their written informed consent to participate in this study. Written informed consent was obtained from the individual(s) for the publication of any potentially identifiable images or data included in this article.

## Author Contributions

RZ contributed to the conceptualization and drafted the original draft preparation. JL conducted the data analysis. RZ, JL, and MC revised the manuscript. All authors contributed to the article and approved the submitted version.

## Conflict of Interest

The authors declare that the research was conducted in the absence of any commercial or financial relationships that could be construed as a potential conflict of interest.

## Publisher’s Note

All claims expressed in this article are solely those of the authors and do not necessarily represent those of their affiliated organizations, or those of the publisher, the editors and the reviewers. Any product that may be evaluated in this article, or claim that may be made by its manufacturer, is not guaranteed or endorsed by the publisher.

## References

[1] World Health Organization. *Ageing, Life Course Unit. WHO Global Report on Falls Prevention in Older Age.* (2008). Available online at: https://www.who.int/publications/i/item/9789241563536 (accessed March 17, 2008).

[2] James SL Lucchesi LR Bisignano C Castle CD Dingels ZV Fox JT The global burden of falls: global, regional and national estimates of morbidity and mortality from the global burden of disease study 2017. *Inj Prev.* (2020) 26:i3–11. 10.1136/injuryprev-2019-043286 31941758 PMC7571347

[3] Gerards MHG McCrum C Mansfield A Meijer K. Perturbation-based balance training for falls reduction among older adults: current evidence and implications for clinical practice. *Geriatr Gerontol Int.* (2017) 17:2294–303. 10.1111/ggi.13082 28621015 PMC5763315

[4] World Health Organization. *WHO Global Report on Falls Prevention in Older Age.* (2007). Available online at: https://www.who.int/ageing/publications/Falls_prevention7March.pdf?ua=1 (accessed March 7, 2007).

[5] Dunn JE Rudberg MA Furner SE Cassel CK. Mortality, disability, and falls in older persons: the role of underlying disease and disability. *Am J Public Health.* (1992) 82:395–400. 10.2105/ajph.82.3.395 1531583 PMC1694374

[6] Sherrington C Michaleff ZA Fairhall N Paul SS Tiedemann A Whitney J Exercise to prevent falls in older adults: an updated systematic review and meta-analysis. *Br J Sports Med.* (2017) 51:1750–8. 10.1136/bjsports-2016-096547 27707740

[7] Diniz BS Lima-Costa MF Peixoto SV Firmino J Torres K Martins-Filho OA Cognitive frailty is associated with elevated proinflammatory markers and a higher risk of mortality (published online ahead of print, 2022 Feb 3). *Am J Geriatr Psychiatry.* (2022) 30:825–33. 10.1016/j.jagp.2022.01.012 35227616 PMC9177532

[8] Tinetti ME Kumar C. The patient who falls: “it’s always a trade-off”. *JAMA.* (2010) 303:258–66. 10.1001/jama.2009.2024 20085954 PMC3740370

[9] Montero-Odasso M Muir SW Speechley M. Dual-task complexity affects gait in people with mild cognitive impairment: the interplay between gait variability, dual tasking, and risk of falls. *Arch Phys Med Rehabil.* (2012) 93:293–9. 10.1016/j.apmr.2011.08.026 22289240

[10] Shaw FE. Falls in cognitive impairment and dementia. *Clin Geriatr Med.* (2002) 18:159–73. 10.1016/S0749-0690(02)00003-4 12180241

[11] Kallin K Gustafson Y Sandman PO Karlsson S. Factors associated with falls among older, cognitively impaired people in geriatric care settings: a population-based study. *Am J Geriatr Psychiatry.* (2005) 13:501–9. 10.1176/appi.ajgp.13.6.501 15956270

[12] Welmer AK Rizzuto D Laukka EJ Johnell K Fratiglioni L. Cognitive and physical function in relation to the risk of injurious falls in older adults: a population-based study. *J Gerontol A Biol Sci Med Sci.* (2017) 72:669–75. 10.1093/gerona/glw141 27449140

[13] Martin KL Blizzard L Srikanth VK Wood A Thomson R Sanders LM Cognitive function modifies the effect of physiological function on the risk of multiple falls–a population-based study. *J Gerontol A Biol Sci Med Sci.* (2013) 68:1091–7. 10.1093/gerona/glt010 23410920

[14] Welmer AK Rizzuto D Qiu C Caracciolo B Laukka EJ. Walking speed, processing speed, and dementia: a population-based longitudinal study. *J Gerontol A Biol Sci Med Sci.* (2014) 69:1503–10. 10.1093/gerona/glu047 24706441

[15] Wang Z Dong B. Screening for cognitive impairment in geriatrics. *Clin Geriatr Med.* (2018) 34:515–36. 10.1016/j.cger.2018.06.004 30336986

[16] Schootemeijer S Weijer RHA Hoozemans MJM van Schooten KS Delbaere K Pijnappels M. Association between daily-life gait quality characteristics and physiological fall risk in older people. *Sensors (Basel).* (2020) 20:5580. 10.3390/s20195580 33003414 PMC7582484

[17] Greene BR McManus K Redmond SJ Caulfield B Quinn CC. Digital assessment of falls risk, frailty, and mobility impairment using wearable sensors. *NPJ Digit Med.* (2019) 2:125. 10.1038/s41746-019-0204-z 31840096 PMC6906412

[18] Yang NP Hsu NW Lin CH Chen HC Tsao HM Lo SS Relationship between muscle strength and fall episodes among the elderly: the Yilan study, Taiwan. *BMC Geriatr.* (2018) 18:90. 10.1186/s12877-018-0779-2 29653515 PMC5899404

[19] Yamada T Demura S. Effectiveness of sit-to-stand tests for evaluating physical functioning and fall risk in community-dwelling elderly. *Hum Perform Meas.* (2015) 12:1–7. 10.14859/hpm.12.1

[20] Simpkins C Yang F. Muscle power is more important than strength in preventing falls in community-dwelling older adults. *J Biomech.* (2022) 134:111018. 10.1016/j.jbiomech.2022.111018 35228153

[21] Wang L Song P Cheng C Han P Fu L Chen X The added value of combined timed up and go test, walking speed, and grip strength on predicting recurrent falls in chinese community-dwelling elderly. *Clin Interv Aging.* (2021) 16:1801–12. 10.2147/CIA.S325930 34675495 PMC8502011

[22] Goh JW Singh DKA Mesbah N Hanafi AAM Azwan AF. Fall awareness behaviour and its associated factors among community dwelling older adults. *BMC Geriatr* (2021) 21:226. 10.1186/s12877-021-02122-z 33823808 PMC8022521

[23] Phelan EA Ritchey K. Fall prevention in community-dwelling older adults. *Ann Intern Med.* (2018) 169:ITC81–96. 10.7326/AITC201812040 30508457

[24] Legrand D Vaes B Matheï C Adriaensen W Van Pottelbergh G Degryse JM. Muscle strength and physical performance as predictors of mortality, hospitalization, and disability in the oldest old. *J Am Geriatr Soc.* (2014) 62:1030–8. 10.1111/jgs.12840 24802886

[25] Zhao Y Hu Y Smith JP Strauss J Yang G. Cohort profile: the China health and retirement longitudinal study (CHARLS). *Int J Epidemiol.* (2014) 43:61–8. 10.1093/ije/dys203 23243115 PMC3937970

[26] Holsinger T Deveau J Boustani M Williams JW. Does this patient have dementia? *JAMA.* (2007) 297:2391–404. 10.1001/jama.297.21.2391 17551132

[27] Essien SK Feng CX Sun W Farag M Li L Gao Y. Sleep duration and sleep disturbances in association with falls among the middle-aged and older adults in China: a population-based nationwide study. *BMC Geriatr.* (2018) 18:196. 10.1186/s12877-018-0889-x 30153791 PMC6114492

[28] Qian J Li N Ren X. Obesity and depressive symptoms among Chinese people aged 45 and over. *Sci Rep.* (2017) 7:45637. 10.1038/srep45637 28378748 PMC5381219

[29] Andresen EM Malmgren JA Carter WB Patrick DL. Screening for depression in well older adults: evaluation of a short form of the CES-D (center for epidemiologic studies depression scale). *Am J Prev Med.* (1994) 10:77–84. 8037935

[30] Kwan MM Close JC Wong AK Lord SR. Falls incidence, risk factors, and consequences in Chinese older people: a systematic review. *J Am Geriatr Soc.* (2011) 59:536–43. 10.1111/j.1532-5415.2010.03286.x 21361880

[31] Chu LW Chi I Chiu AY. Incidence and predictors of falls in the chinese elderly. *Ann Acad Med Singap.* (2005) 34:60–72. 15726221

[32] Ganz DA Latham NK. Prevention of falls in community-dwelling older adults. *N Engl J Med.* (2020) 382:734–43. 10.1056/NEJMcp1903252 32074420

[33] Campbell AJ Borrie MJ Spears GF. Risk factors for falls in a community-based prospective study of people 70 years and older. *J Gerontol.* (1989) 44:M112–7. 10.1093/geronj/44.4.m112 2738307

[34] Zhao YL Alderden J Lind B Stibrany J. Risk factors for falls in homebound community-dwelling older adults. *Public Health Nurs.* (2019) 36:772–8. 10.1111/phn.12651 31407384

[35] Wu L Sun D. Sleep duration and falls: a systemic review and meta-analysis of observational studies. *J Sleep Res.* (2017) 26:293–301. 10.1111/jsr.12505 28220576

[36] Zhang Q Wu Y Han T Liu E. Changes in cognitive function and risk factors for cognitive impairment of the elderly in China: 2005-2014. *Int J Environ Res Public Health.* (2019) 16:2847. 10.3390/ijerph16162847 31404951 PMC6719934

[37] Deandrea S Lucenteforte E Bravi F Foschi R La Vecchia C Negri E. Risk factors for falls in community-dwelling older people: a systematic review and meta-analysis. *Epidemiology.* (2010) 21:658–68. 10.1097/EDE.0b013e3181e89905 20585256

[38] Muir SW Gopaul K Montero Odasso MM. The role of cognitive impairment in fall risk among older adults: a systematic review and meta-analysis. *Age Ageing.* (2012) 41:299–308. 10.1093/ageing/afs012 22374645

[39] Ma Y Li X Pan Y Zhao R Wang X Jiang X Cognitive frailty and falls in Chinese elderly people: a population-based longitudinal study. *Eur J Neurol.* (2021) 28:381–8. 10.1111/ene.14572 33030300

[40] Cöster ME Karlsson M Ohlsson C Mellström D Lorentzon M Ribom E Physical function tests predict incident falls: a prospective study of 2969 men in the Swedish osteoporotic fractures in men study. *Scand J Public Health.* (2020) 48:436–41. 10.1177/1403494818801628 30269679

[41] Wickham C Cooper C Margetts BM Barker DJ. Muscle strength, activity, housing and the risk of falls in elderly people. *Age Ageing.* (1989) 18:47–51. 10.1093/ageing/18.1.47 2565665

[42] Miller MD Giles LC Crotty M Harrison JE Andrews GR. A clinically relevant criterion for grip strength: relationship with falling in a sample of older adults. *Nutr Diet.* (2003) 60:248–53.

[43] Verghese J Robbins M Holtzer R Zimmerman M Wang C Xue X Gait dysfunction in mild cognitive impairment syndromes. *J Am Geriatr Soc.* (2008) 56:1244–51. 10.1111/j.1532-5415.2008.01758.x 18482293 PMC2574944

[44] Louis ED Schupf N Manly J Marder K Tang MX Mayeux R. Association between mild parkinsonian signs and mild cognitive impairment in a community. *Neurology.* (2005) 64:1157–61. 10.1212/01.WNL.0000156157.97411.5E 15824340

[45] Liu-Ambrose TY Ashe MC Graf P Beattie BL Khan KM. Increased risk of falling in older community-dwelling women with mild cognitive impairment. *Phys Ther.* (2008) 88:1482–91. 10.2522/ptj.20080117 18820094 PMC3514550

[46] Taylor ME Delbaere K Lord SR Mikolaizak AS Close JC. Physical impairments in cognitively impaired older people: implications for risk of falls. *Int Psychogeriatr.* (2013) 25:148–56. 10.1017/S1041610212001184 22831907

[47] Clouston SA Brewster P Kuh D Richards M Cooper R Hardy R The dynamic relationship between physical function and cognition in longitudinal aging cohorts. *Epidemiol Rev.* (2013) 35:33–50. 10.1093/epirev/mxs004 23349427 PMC3578448

[48] Zeng Y Feng Q Hesketh T Christensen K Vaupel J. Improvements in survival and activities of daily living despite declines in physical and cognitive functioning among the oldest-old in China–evidence from a cohort study. *Lancet.* (2017) 389:1619–29. 10.1016/S0140-6736(17)30548-2 28285816 PMC5406246

[49] Park SH Lee YS. The diagnostic accuracy of the berg balance scale in predicting falls. *West J Nurs Res.* (2017) 39:1502–25. 27784833 10.1177/0193945916670894

[50] Park SH. Tools for assessing fall risk in the elderly: a systematic review and meta-analysis. *Aging Clin Exp Res.* (2018) 30:1–16. 10.1007/s40520-017-0749-0 28374345

[51] Alcazar J Losa-Reyna J Rodriguez-Lopez C Alfaro-Acha A Rodriguez-Mañas L Ara I The sit-to-stand muscle power test: an easy, inexpensive and portable procedure to assess muscle power in older people. *Exp Gerontol.* (2018) 112:38–43. 10.1016/j.exger.2018.08.006 30179662

[52] Phirom K Kamnardsiri T Sungkarat S. Beneficial effects of interactive physical-cognitive game-based training on fall risk and cognitive performance of older adults. *Int J Environ Res Public Health.* (2020) 17:6079. 10.3390/ijerph17176079 32825555 PMC7504204

