# Supplementary material for: The Value of Cognitive and Physical Function Tests in Predicting Falls in Older Adults: A Prospective Study
Source: Front Med (Lausanne). 2022 Jul 5;9:900488. doi: 10.3389/fmed.2022.900488 (PMC9294354; doi:10.3389/fmed.2022.900488)
Supplement: Supplementary file 1 [file Table_1.DOCX]

Supplementary Material

**Supplementary Table 1 |** The results of the Pearson correlation test

| **Education level** | **Pearson** | 1 | -0.273 | -0.063 | -0.088 | -0.117 | 0.035 | 0.047 | 0.047 | 0.022 | 0.085 | 0.073 | 0.033 | -0.025 | 0.015 | 0.072 |
| --- | --- | --- | --- | --- | --- | --- | --- | --- | --- | --- | --- | --- | --- | --- | --- | --- |
|  | ***p*** |  | <0.001 | <0.001 | <0.001 | <0.001 | 0.014 | 0.001 | 0.001 | 0.126 | <0.001 | <0.001 | 0.021 | 0.082 | 0.286 | <0.001 |
| **Gender** | **Pearson** | -0.273 | 1 | -0.068 | 0.123 | 0.547 | -0.110 | -0.112 | 0.045 | 0.018 | -0.041 | 0.040 | 0.004 | 0.096 | 0.059 | -0.090 |
|  | ***p*** | <0.001 |  | <0.001 | <0.001 | <0.001 | <0.001 | <0.001 | 0.002 | 0.198 | 0.004 | 0.005 | 0.79 | <0.001 | <0.001 | <0.001 |
| **Age** | **Pearson** | -0.063 | -0.068 | 1 | 0.192 | 0.011 | -0.025 | 0.038 | -0.001 | -0.005 | -0.071 | -0.146 | 0.003 | 0.004 | 0.02 | -0.033 |
|  | ***p*** | <0.001 | <0.001 |  | <0.001 | 0.444 | 0.078 | 0.008 | 0.929 | 0.749 | <0.001 | <0.001 | 0.82 | 0.794 | 0.163 | 0.021 |
| **Marital status** | **Pearson** | -0.088 | 0.123 | 0.192 | 1 | 0.036 | -0.040 | -0.009 | -0.023 | -0.019 | -0.045 | -0.050 | -0.039 | 0.031 | -0.01 | -0.023 |
|  | ***p*** | <0.001 | <0.001 | <0.001 |  | 0.012 | 0.005 | 0.542 | 0.107 | 0.19 | 0.002 | <0.001 | 0.006 | 0.031 | 0.489 | 0.103 |
| **smoking** | **Pearson** | -0.117 | 0.547 | 0.011 | 0.036 | 1 | -0.069 | -0.047 | 0.015 | 0.008 | -0.02 | 0.004 | 0.012 | 0.056 | 0.083 | -0.031 |
|  | ***p*** | <0.001 | <0.001 | 0.444 | 0.012 |  | <0.001 | 0.001 | 0.298 | 0.56 | 0.163 | 0.757 | 0.417 | <0.001 | <0.001 | 0.032 |
| **Night sleep duration** | **Pearson** | 0.035 | -0.110 | -0.025 | -0.040 | -0.069 | 1 | 0.122 | 0.049 | 0.046 | 0.097 | 0.051 | 0.017 | -0.115 | -0.081 | 0.122 |
|  | ***p*** | 0.014 | <0.001 | 0.078 | 0.005 | <0.001 |  | <0.001 | 0.001 | 0.001 | <0.001 | <0.001 | 0.225 | <0.001 | <0.001 | <0.001 |
| **Nap duration** | **Pearson** | 0.047 | -0.112 | 0.038 | -0.009 | -0.047 | 0.122 | 1 | 0.011 | 0.008 | 0.030 | 0.004 | -0.029 | -0.024 | 0.005 | 0.042 |
|  | ***p*** | 0.001 | <0.001 | 0.008 | 0.542 | 0.001 | <0.001 |  | 0.426 | 0.563 | 0.039 | 0.763 | 0.042 | 0.097 | 0.731 | 0.003 |
| **Physical disabilities** | **Pearson** | 0.047 | 0.045 | -0.001 | -0.023 | 0.015 | 0.049 | 0.011 | 1 | 0.133 | 0.105 | 0.062 | 0.004 | -0.032 | -0.069 | 0.070 |
|  | ***p*** | 0.001 | 0.002 | 0.929 | 0.107 | 0.298 | 0.001 | 0.426 |  | <0.001 | <0.001 | <0.001 | 0.754 | 0.025 | <0.001 | <0.001 |
| **Intellectual disability** | **Pearson** | 0.022 | 0.018 | -0.005 | -0.019 | 0.008 | 0.046 | 0.008 | 0.133 | 1 | 0.112 | 0.113 | 0.103 | -0.050 | -0.074 | 0.062 |
|  | ***p*** | 0.126 | 0.198 | 0.749 | 0.19 | 0.56 | 0.001 | 0.563 | <0.001 |  | <0.001 | <0.001 | <0.001 | <0.001 | <0.001 | <0.001 |
| **Vision problem** | **Pearson** | 0.085 | -0.041 | -0.071 | -0.045 | -0.02 | 0.097 | 0.030 | 0.105 | 0.112 | 1 | 0.192 | 0.040 | -0.049 | -0.091 | 0.043 |
|  | ***p*** | <0.001 | 0.004 | <0.001 | 0.002 | 0.163 | <0.001 | 0.039 | <0.001 | <0.001 |  | <0.001 | 0.006 | 0.001 | <0.001 | 0.003 |
| **Hearing problem** | **Pearson** | 0.073 | 0.040 | -0.146 | -0.050 | 0.004 | 0.051 | 0.004 | 0.062 | 0.113 | 0.192 | 1 | 0.061 | -0.032 | -0.066 | 0.065 |
|  | ***p*** | <0.001 | 0.005 | <0.001 | <0.001 | 0.757 | <0.001 | 0.763 | <0.001 | <0.001 | <0.001 |  | <0.001 | 0.027 | <0.001 | <0.001 |
| **Speech impediment** | **Pearson** | 0.033 | 0.004 | 0.003 | -0.039 | 0.012 | 0.017 | -0.029 | 0.004 | 0.103 | 0.040 | 0.061 | 1 | -0.024 | -0.011 | 0.01 |
|  | ***p*** | 0.021 | 0.79 | 0.82 | 0.006 | 0.417 | 0.225 | 0.042 | 0.754 | <0.001 | 0.006 | <0.001 |  | 0.096 | 0.437 | 0.48 |
| **CES-D** | **Pearson** | -0.025 | 0.096 | 0.004 | 0.031 | 0.056 | -0.115 | -0.024 | -0.032 | -0.050 | -0.049 | -0.032 | -0.024 | 1 | 0.063 | -0.047 |
|  | ***p*** | 0.082 | <0.001 | 0.794 | 0.031 | <0.001 | <0.001 | 0.097 | 0.025 | <0.001 | 0.001 | 0.027 | 0.096 |  | <0.001 | 0.001 |
| **Chronic diseases** | **Pearson** | 0.015 | 0.059 | 0.02 | -0.01 | 0.083 | -0.081 | 0.005 | -0.069 | -0.074 | -0.091 | -0.066 | -0.011 | 0.063 | 1 | -0.071 |
|  | ***p*** | 0.286 | <0.001 | 0.163 | 0.489 | <0.001 | <0.001 | 0.731 | <0.001 | <0.001 | <0.001 | <0.001 | 0.437 | <0.001 |  | <0.001 |
| **History of falls** | **Pearson** | 0.072 | -0.090 | -0.033 | -0.023 | -0.031 | 0.122 | 0.042 | 0.070 | 0.062 | 0.043 | 0.065 | 0.01 | -0.047 | -0.071 | 1 |
|  | *p* | <0.001 | <0.001 | 0.021 | 0.103 | 0.032 | <0.001 | 0.003 | <0.001 | <0.001 | 0.003 | <0.001 | 0.48 | 0.001 | <0.001 |  |
|  |  | Education level | Gender | Age | Marital status | Smoking | Night sleep duration | Nap duration | Physical disabilities | Intellectual disability | Vision problem | Hearing problem | Speech impediment | CES-D | Chronic diseases | History of falls |
